# Sialylation Inhibition Can Partially Revert Acquired Resistance to Enzalutamide in Prostate Cancer Cells

**DOI:** 10.3390/cancers16172953

**Published:** 2024-08-24

**Authors:** Emily Archer Goode, Margarita Orozco-Moreno, Kirsty Hodgson, Amirah Nabilah, Meera Murali, Ziqian Peng, Jona Merx, Emiel Rossing, Johan F. A. Pijnenborg, Thomas J. Boltje, Ning Wang, David J. Elliott, Jennifer Munkley

**Affiliations:** 1 Newcastle University Centre for Cancer, Newcastle University Institute of Biosciences, Newcastle NE1 3BZ, UK; e.archer-goode@newcastle.ac.uk (E.A.G.); maggie.orozco-moreno@newcastle.ac.uk (M.O.-M.); kirsty.hodgson@newcastle.ac.uk (K.H.); a.nabilah2@newcastle.ac.uk (A.N.); m.murali2@newcastle.ac.uk (M.M.); z.peng9@newcastle.ac.uk (Z.P.); david.elliott@newcastle.ac.uk (D.J.E.); 2 Synthetic Organic Chemistry, Institute for Molecules and Materials, Radboud University, 6525 XZ Nijmegen, The Netherlands; jona.merx@ru.nl (J.M.); thomas.boltje@ru.nl (T.J.B.); 3 GlycoTherapeutics B.V., 6511 AJ Nijmegen, The Netherlands; emiel.rossing@glycotherapeutics.eu (E.R.); johan.pijnenborg@glycotherapeutics.eu (J.F.A.P.); 4 The Mellanby Centre for Musculoskeletal Research, Division of Clinical Medicine, The University of Sheffield, Sheffield S10 2TN, UK; nw208@leicester.ac.uk; 5 Leicester Cancer Research Centre, Department of Genetics and Genome Biology, University of Leicester, Leicester LE2 7LX, UK

**Keywords:** prostate cancer, castrate resistance, enzalutamide, sialic acid, sialyltransferase inhibitor, combination therapies

## Abstract

**Simple Summary:**

Prostate cancer is the most common cancer in men and a major cause of cancer-related deaths around the world. Prostate cancer that has spread to other parts of the body (advanced prostate cancer) is often treated with a drug called enzalutamide, which is a type of hormone therapy. Enzalutamide works by blocking the effect of the testosterone hormone on prostate cancer cells to stop them from growing. While this can be effective for several years, unfortunately, many patients being treated with enzalutamide eventually go on to become resistant to treatment, and the therapy stops working. Here, we show that prostate cancer cells that have become resistant to enzalutamide have increased levels of a type of sugar (known as sialic acid) on their surfaces. We set out to test whether stripping sialic acid from the surface of prostate cancer cells could help keep enzalutamide working for longer. Excitingly, our experiments show that treating prostate cancer cells with a drug to block sialic acid can partially reverse enzalutamide resistance. These findings suggest that drugs targeting sialic acid could be used in combination with enzalutamide therapy to disarm drug resistance and provide urgently needed new treatment options for men with prostate cancer.

**Abstract:**

Prostate cancer is a lethal solid malignancy and a leading cause of cancer-related deaths in males worldwide. Treatments, including radical prostatectomy, radiotherapy, and hormone therapy, are available and have improved patient survival; however, recurrence remains a huge clinical challenge. Enzalutamide is a second-generation androgen receptor antagonist that is used to treat castrate-resistant prostate cancer. Among patients who initially respond to enzalutamide, virtually all acquire secondary resistance, and an improved understanding of the mechanisms involved is urgently needed. Aberrant glycosylation, and, in particular, alterations to sialylated glycans, have been reported as mediators of therapy resistance in cancer, but a link between tumour-associated glycans and resistance to therapy in prostate cancer has not yet been investigated. Here, using cell line models, we show that prostate cancer cells with acquired resistance to enzalutamide therapy have an upregulation of the sialyltransferase ST6 beta-galactoside alpha-2,6-sialyltransferase 1 (ST6GAL1) and increased levels of α2,6-sialylated *N*-glycans. Furthermore, using the sialyltransferase inhibitor P-SiaFNEtoc, we discover that acquired resistance to enzalutamide can be partially reversed by combining enzalutamide therapy with sialic acid blockade. Our findings identify a potential role for ST6GAL1-mediated aberrant sialylation in acquired resistance to enzalutamide therapy for prostate cancer and suggest that sialic acid blockade in combination with enzalutamide may represent a novel therapeutic approach in patients with advanced disease. Our study also highlights the potential to bridge the fields of cancer biology and glycobiology to develop novel combination therapies for prostate cancer.

## 1. Introduction

Prostate cancer is the second most common cancer in men worldwide and is a significant cause of morbidity and mortality in the global male population [1]. Although localised prostate cancer is largely curable and has a five-year survival rate of >99%, the mortality rate for advanced prostate cancer is high, and only 32% of advanced prostate cancer patients will be alive after 5 years [2]. Primary prostate cancer is largely driven through androgen signalling via interactions with the androgen receptor (AR) and is commonly therapeutically managed with androgen deprivation therapies (ADT) [3,4]. Although ADT achieves initial success, the majority of patients will develop resistance to these therapies within 5 years of diagnosis and will go on to develop castrate-resistant prostate cancer (CRPC), where tumours persist despite low androgen conditions due to the acquisition of resistance mechanisms [5,6]. Patients with CRPC are managed with second-generation androgen receptor inhibitors (enzalutamide, abiraterone, apalutamide, or darolutamide), radium-233 for bone metastases, immunotherapy (sipuleucel-T, pembrolizumab), poly-ADP ribose polymerase (PARP) inhibitors (olaparib, rucaparib), or chemotherapy (most commonly docetaxel) [2,7,8,9,10,11]. However, resistance to these interventions is commonplace, culminating in the dire median survival of 9–30 months for CRPC patients [2,12].

Enzalutamide (MDV3100, Xtandi^®^) is a commonly used second-generation AR inhibitor that demonstrates a higher affinity for binding to the AR in comparison to its predecessors, such as bicalutamide, and has the capacity to prevent AR nuclear translocation, DNA-binding, and recruitment of co-activators [13,14,15,16,17,18]. Clinical trial data have highlighted that the inclusion of enzalutamide in the clinical regimen for treating patients with CRPC could significantly improve overall survival and progression-free survival [13,15,19,20,21]. However, both primary and acquired resistance are observed in relation to enzalutamide treatment. Primary resistance to enzalutamide (defined as treatment failure within the first 3 months following treatment initiation) occurs in 25% of CRPC patients, and acquired resistance is typically observed 9–15 months following treatment initiation [14,22,23,24]. How CRPC tumours develop resistance to enzalutamide remains to be fully understood, but it likely includes AR amplification, AR mutations, the generation of AR splice variants (AR-v), alterations to steroidogenesis, overexpression of glucocorticoid and progesterone receptors, and neuroendocrine differentiation [14,22,25]. As most patients treated with enzalutamide eventually develop resistance and disease progression, there is a critical need to identify mechanisms of resistance as targets for future therapies [26,27]

Glycosylation is a post translational modification where glycan structures are added to proteins and lipids [28,29,30]. Altered glycosylation is a hallmark of cancer and can mediate critical events in tumour development and progression [31,32]. Even though aberrant glycosylation is a potentially druggable hallmark of cancer [28,33,34,35], to date, it has been relatively underexplored particularly in the context of prostate cancer. Studies have sought to understand the molecular mechanisms underpinning prostate cancer progression and resistance to therapy [36,37,38,39,40], but only a few have studied glycans. Historically, this has been due to technological limitations, but this is now changing. For example, the development of *N*-glycan imaging mass spectrometry [41,42,43] has enabled the profiling of glycosylation changes throughout prostate cancer evolution [44]. An understanding of the role glycans play in prostate cancer will be crucial to the identification of new targets that can be exploited to treat advanced disease and prolong patient survival [45,46,47,48]. Recent studies have highlighted the potential to target aberrant glycosylation in combination with existing therapies to boost treatment response and potentially overcome therapy resistance [34,49,50] and this area of research is beginning to show promise for prostate cancer [51,52,53,54].

A common change in tumour glycosylation is alterations to sialylated glycans, and this can actively drive aggressive disease [55,56,57,58]. Aberrant sialylation has been linked to paclitaxel and cisplatin resistance in ovarian cancer [59,60,61], chemotherapy resistance in gastric cancer [62], sensitivity to docetaxel in hepatocarcinoma [63], multi-drug resistance in leukaemia [64,65], sensitivity to tyrosine kinase inhibition in lung cancer [66], and bortezomib resistance in myeloma [67]. A correlation between radiotherapy resistance and increased sialylation is also well established, particularly for colorectal cancer [68,69,70,71]. The contribution of sialylated glycans to chemotherapy resistance in cancer could be due to the physical barrier of extra sialic acid on the surface of cells which can potentially modify key receptors or block the uptake of drugs into the cell. For example, sialylation of the oncogenic receptor Erb2 can mask an epitope recognised by the anti-cancer antibody trastuzumab and promote resistance [72]. Sialylated glycans may also promote therapy resistance by absorbing ionising radiation [56], and radiation exposure can enhance the sialylation of membrane glycoproteins to promote radiation resistance [68,73]. Taken together, these studies raise the possibility of targeting aberrant sialylation in combination with existing cancer therapies to improve patient outcomes.

In prostate cancer, a rewiring of the tumour glycome is associated with disease progression, and glycans play important roles in tumour growth, metastasis, and immune evasion [45,48,74,75,76]. Recently, we showed that ST6 beta-galactoside alpha-2,6-sialyltransferase 1 (ST6GAL1) and larger branched sialylated *N*-glycans are upregulated in men with prostate cancer, and this can promote tumour growth and the spread of tumours to bone [52,77]. Aberrant sialylation is causally linked to therapy resistance in cancer [55], and ST6GAL1 has been identified as a mediator of treatment resistance in several tumour types, including pancreatic cancer [78], colorectal cancer [73,79], leukaemia [80,81], and gastric cancer [72]. A previous study showed resistance to hormonal therapy in prostate cancer is associated with an upregulation of complex larger-branched *N*-glycans [74]. However, to date, studies investigating the role of ST6GAL1 and its associated glycans in therapy-resistant prostate cancer are lacking.

Here, using VCaP and LNCaP cell line models, we show that prostate cancer cells with acquired resistance to enzalutamide have upregulated ST6GAL1 and significantly higher levels of α2-6 sialylated *N*-glycans. Our findings identify ST6GAL1-mediated aberrant sialylation as a potential mediator of acquired resistance to enzalutamide therapy in prostate cancer. Furthermore, using the newly developed C-5 carbamate sialyltransferase inhibitor P-SiaFNEtoc [82,83], we show that acquired resistance to enzalutamide by prostate cancer cells can be partially reversed by sialic acid blockade. These results suggest that inhibiting sialylation in combination with enzalutamide may represent a novel therapeutic approach in patients with advanced prostate cancer.

## 2. Methods

### 2.1. Cell Culture

Cell culture was carried out as described previously [84]. VCaP (CRL-2876) and LNCaP (CRL-1740) cells were purchased from ATCC. Enzalutamide-resistant VCaP (VCaP^EnzR^) and LNCaP (LNCaP^EnzR^) cell lines were generated as described previously [85]. Briefly, LNCaP and VCaP cells were grown in 10 μM enzalutamide in long-term culture (>6 months). Resistant cells were pooled and maintained. Once resistant, cells were continually cultured in 10 μM enzalutamide. No changes in phenotype were observed, and we did not detect any genomic changes. The cell lines were authenticated using DNA STR analysis and tested every 3 months for mycoplasma contamination.

### 2.2. Inhibitors

The enzalutamide (MDV3100, Xtandi^®^) was purchased from MedChemExpress^®^ (HY-70002). The sialyltransferase inhibitor P-SiaFNEtoc was synthesised as described previously [82] (compound 10). 

### 2.3. Western Blotting

Western blotting was performed as previously described [86]. Immunoblots were probed with antibodies for ST6GAL1 at 1:1000 dilution (Abgent, San Diego, CA, USA, AP19891c) or GAPDH at 1:2000 dilution (Abgent, AP7873b), followed by incubation with appropriate fluorescent secondary antibodies at 1:10,000 dilution, (anti-mouse 680, Cell Signalling, Leiden, The Netherlands, 5470S) or anti-rabbit 800 (Cell Signalling, 5151S)).

### 2.4. ELISA Assays

Conditioned media samples were prepared from cell lines as described previously [86]. ST6GAL1 protein levels were monitored using sandwich ELISA assays, which have previously been validated (Cambridge Bioscience, Cambridge, UK, ELH-ST6GAL1-1) [77].

### 2.5. Immunocytochemistry

The cells were cultured in a Nunc™ 4 well plate (Thermo Scientific™, Oxford, UK, 176740) on top of a sterilised 10 mm-round coverslip (VWRTM, 631-1340) in complete media. Treatments with P-SiaFNEtoc were performed for either for 3 days (LNCaP cells) or 6 days (VCaP cells) with the indicated concentrations. Cells treated with DMSO were used as controls. The cells were washed with PBS before permeabilization and fixation with ice-cold absolute methanol for 10 min at −20 °C. Next, the cells were washed with PBS and blocked with 10% goat serum (Abcam, Cambridge, UK, ab7481) for 1 h at room temperature. The cells were incubated overnight at 4 °C with a ST6GAL1 antibody at 1:200 (Abgent, AP19891c), followed by goat anti-rabbit IgG H and L (Alexa Fluor^®^ 594) (Abcam, ab150080), diluted 1:500. Finally, the cells were washed with PBS and stained with Hoechst (Thermo Scientific, 62249) for 15 min at room temperature. Images were acquired and processed with a ZEISS Axio Imager 4. 

### 2.6. Lectin Immunofluorescence

The cells were cultured in a Nunc™ 4-well plate (Thermo Scientific™, 176740) on top of a sterilised 10 mm-round coverslip (VWRTM, 631-1340) in complete media. Treatments with P-SiaFNEtoc were performed for either for 3 days (LNCaP cells) or 6 days (VCaP cells) with the indicated concentrations. Cells treated with DMSO were used as controls. P-SiaFNEtoc concentrations were optimised to 2 µM for 3 days and 20 µM for 6 days for the LNCaP and VCaP cells, respectively. For neuraminidase treatment, α2-3,6,8 neuraminidase (NEB, UK, P0720) was used as a negative control to strip sialic acid from the surface of the cells. The cells were cultured in 100 units/mL of neuraminidase as described previously [44]. To monitor Sambucas nigra (SNA) lectin binding, the cells were washed with PBS before permeabilization and fixation with ice-cold absolute methanol for 10 min at −20 °C. Next, the cells were washed with PBS and blocked with 1X Carbo-Free™ Blocking Solution (1X CFB) (Vector Laboratories, Cambs, UK, SP-5040-125) for 1 h at room temperature. The cells were incubated overnight at 4 °C with FITC-conjugated SNA lectin (Vector Laboratoriesabs, FL-1301-2). Finally, the cells were washed with PBS and stained with Hoechst (Thermo Scientific, 62249) for 15 min at room temperature. The cells were mounted using ProLongTM gold antifade mountant (Thermo Scientific™, P36930), and images were acquired and processed with the ZEISS Axio Imager 4.

### 2.7. CellTiter-Glo^®^ Assays

CellTiter-Glo^®^ Luminescent cell viability assays (Promega, UK, G9682) were performed as described previously [51]. Cell viability was assessed at the times indicated, and luminescence was recorded with the Varioskan™ LUX microplate reader. 

VCaP cells: The VCaP control and VCaP^EnzR^ cells were seeded at a density of 7.0 × 10^3^ cells/well in 96-well plates (Thermo cientific, 130188). After 24 h, the cells were treated with a range of concentrations of enzalutamide with or without concurrent treatment with 20 µM P-SiaFNEtoc for 6 days (144 h).

LNCaP cells: The LNCaP control and LNCaP^EnzR^ cells were seeded at a density of 3.0 × 10^3^ cells/well in a 96-well plate. After 24 h, the cells were treated with a range of enzalutamide concentrations with or without concurrent treatment with 2 µM P-SiaFNEtoc for 3 days (72 h).

### 2.8. Statistical Analyses

Statistical analyses were conducted using the GraphPad Prism software (version Prism 9.4.1). Data are presented as the mean of three independent samples ± standard error of the mean (SEM). Statistical significance is denoted as * *p* < 0.05, ** *p* < 0.01, *** *p* < 0.001, and **** *p* < 0.0001. 

## 3. Results

### 3.1. Prostate Cancer Cells with Acquired Enzalutamide Resistance Have Upregulated ST6GAL1 

**Figure 1:**
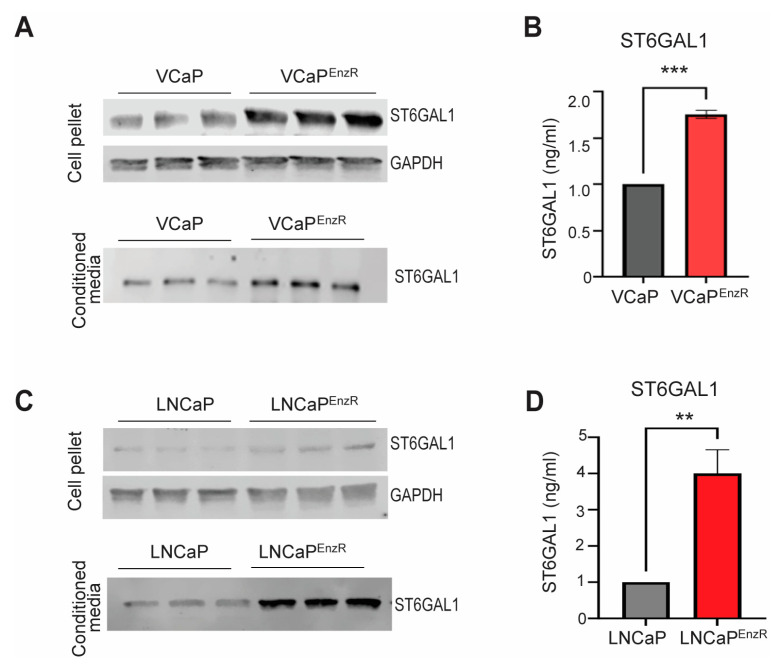
Enzalutamide-resistant prostate cancer cells have upregulation of ST6GAL1. (**A**) Western blot analysis of ST6GAL1 in the VCaP control and enzalutamide-resistant VCaP (VCaP^EnzR^) cells. ST6GAL1 levels are increased in both cell pellet and conditioned media samples from the VCaP^EnzR^ cells. GAPDH is included as a loading control. (**B**) Analysis of ST6GAL1 levels in conditioned media samples from the VCaP control and VCaP^EnzR^ cells using pre-validated sandwich ELISA assays [77]. The levels of ST6GAL1 are significantly higher in conditioned media from VCaP^EnzR^ cells (n = 3, unpaired *t*-test, *** *p* = 0.0002). (**C**) Western blot analysis of ST6GAL1 in the LNCaP control and enzalutamide-resistant LNCaP (LNCaP^EnzR^) cells. GAPDH is used as a loading control. The levels of ST6GAL1 are increased in both cell pellet and conditioned media samples (**D**) Analysis of ST6GAL1 levels in conditioned media samples from the LNCaP control and LNCaP^EnzR^ cells using pre-validated sandwich ELISA assays [77]. The levels of ST6GAL1 are significantly higher in conditioned media from the LNCaP^EnzR^ cells compared to the control LNCaP cells (n = 6, unpaired *t*-test, ** *p* = 0.0014). Results are representative of three biological repeats and are presented as the mean ± standard error. Original western blots are presented in File S1.

The sialyltransferase ST6GAL1 has been previously identified to be upregulated in prostate cancer and linked with tumour growth, metastasis, and poor overall survival [52,77,87,88]. ST6GAL1 has been reported as a mediator of therapy resistance in other cancer types [72,73,78,79,80,81], but a link between ST6GAL1 and resistance to therapy has not yet been investigated for prostate cancer. To address this gap, we used western blotting and pre-validated sandwich ELISA assays [77] to monitor ST6GAL1 protein levels in VCaP and LNCaP prostate cancer cell line models with acquired resistance to enzalutamide. Our findings show intra-cellular and secreted ST6GAL1 levels are upregulated in the enzalutamide-resistant VCaP cells (VCaP^EnzR^), compared to the enzalutamide-sensitive control VCaP cells (Figure 1A,B). Upregulation of ST6GAL1 was also detected in the enzalutamide-resistant LNCaP cells (LNCaP^EnzR^), where levels of ST6GAL1 are increased compared to the enzalutamide-sensitive control LNCaP cells (Figure 1C,D). Taken together, these findings suggest that the sialyltransferase ST6GAL1 is upregulated in prostate cancer cells with acquired resistance to enzalutamide therapy.

### 3.2. Enzalutamide-Resistant Prostate Cancer Cells Have Increased Levels of α2,6-Sialylated N-Glycans

**Figure 2:**
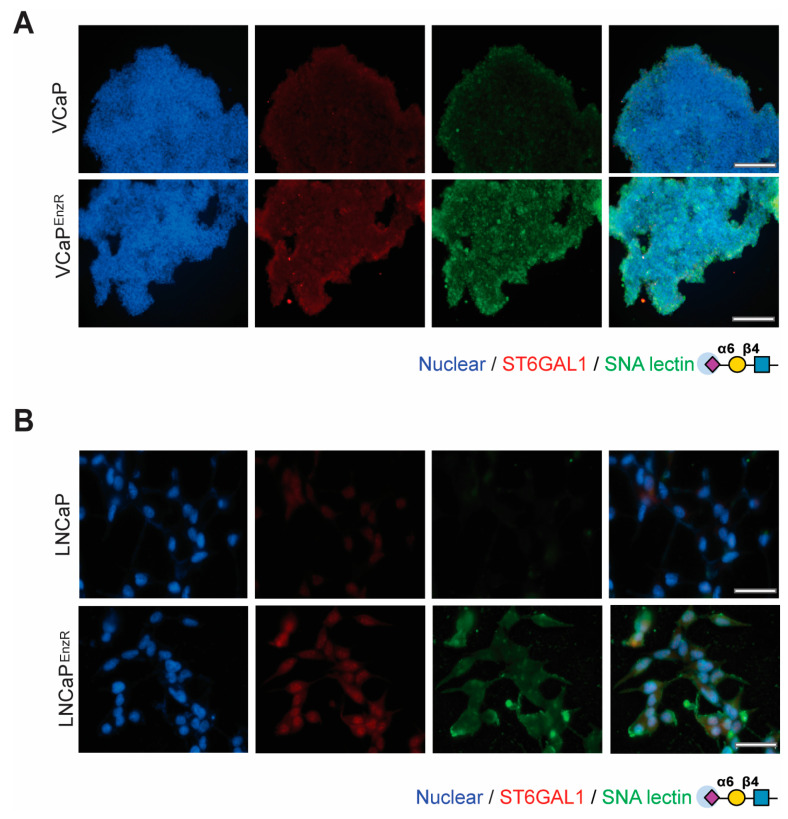
Enzalutamide-resistant prostate cancer cells have upregulation of ST6GAL1 and increased levels of α2,6-sialylated glycans. (**A**) SNA lectin immunofluorescence shows VCaP^EnzR^ cells have increased levels of ST6GAL1 and α2-6 sialylation (SNA, the lectin from Sambucus nigra, recognises α2-6-linked sialylated N-glycans [91]). (**B**) SNA lectin immunofluorescence shows LNCaP^EnzR^ cells have increased levels of ST6GAL1 and α2-6 sialylation compared to control LNCaP cells. DNA is stained with Hoechst. Scale bar = 200 µM.

ST6GAL1 adds α2,6-linked sialic acid to *N*-glycosylated proteins that are destined for the cell membrane or secretion [55,89,90]. In previous studies, we detected increased levels of α2,6 sialylation in prostate cancer cells with an upregulation of ST6GAL1 [52,77]. We thus hypothesised that the upregulation of ST6GAL1 in enzalutamide-resistant prostate cancer cells will alter the levels of α2,6 sialylated *N*-glycans. To test this, we used immunofluorescence to monitor recognition by the SNA lectin (which recognises α2,6-linked sialylated *N*-glycans [91]) in prostate cancer cells with acquired resistance to enzalutamide. Our findings show that the VCaP^EnzR^ cells have increased binding of SNA lectin relative to the control enzalutamide-sensitive VCaP cells (Figure 2A). Similarly, the LNCaP^EnzR^ cells also show increased recognition by SNA lectin, indicating increased levels of α2,6 sialylation in these cells compared to the control enzalutamide-sensitive LNCaP cells (Figure 2B). Confirming the specificity of our results, SNA binding was eliminated when cells were treated with neuraminidase (which removes terminal sialic acids of glycans) (Supplementary Figure S1). In summary, these findings suggest enzalutamide resistance correlates with an upregulation of α2,6-sialylated *N*-glycans in prostate cancer cell line models.

### 3.3. The Sialyltransferase Inhibitor P-SiaFNEtoc Blocks α2,6 Sialylation in Enzalutamide-Resistant Prostate Cancer Cells

**Figure 3:**
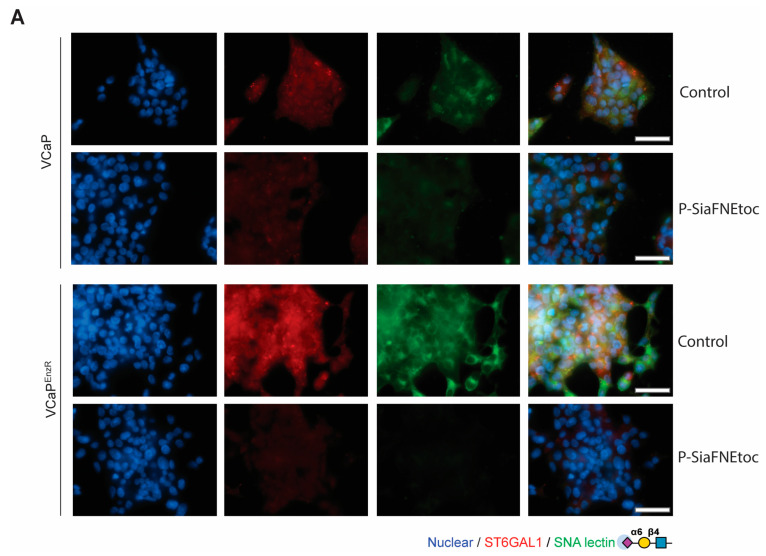

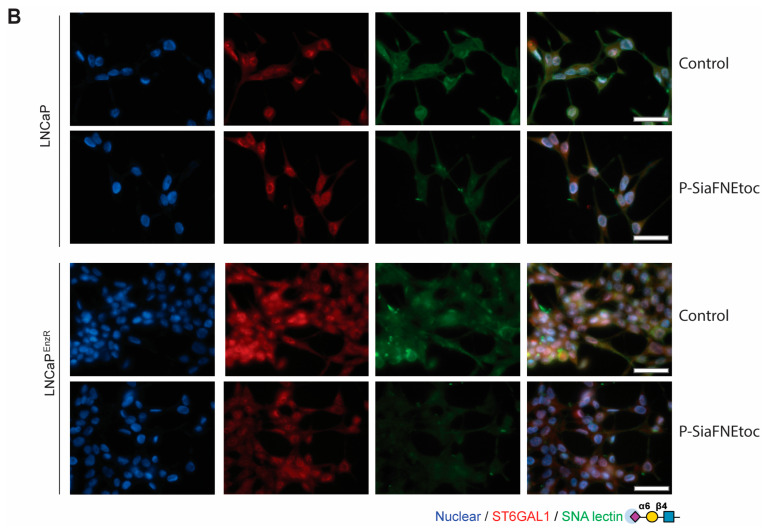
The sialyltransferase inhibitor P-SiaFNEtoc blocks α2,6 sialylation in the VCaP^EnzR^ and LNCaP^EnzR^ prostate cancer cells. (**A**) Detection of immunofluorescent staining of ST6GAL1 and α2,6-sialylation of *N*-glycans in the VCaP control and VCaP^EnzR^ cells treated with 20 µM of the sialyltransferase inhibitor P-SiaFNEtoc for 6 days. Treatment of both cell lines with P-SiaFNEtoc inhibits α2,6-sialylation of *N*-glycans (detected using SNA lectin). Control cells were treated with DMSO. Scale bar = 50 µm. The images are representative of three biological repeats. (**B**) Detection of immunofluorescent staining of ST6GAL1 and α2,6-sialylation of *N*-glycans in LNCaP control and LNCaP^EnzR^ cells treated with 2 µM of the sialyltransferase inhibitor P-SiaFNEtoc for 3 days. Treatment of both cell lines with P-SiaFNEtoc inhibits α2,6-sialylation of *N*-glycans (detected using SNA lectin). The control cells were treated with DMSO. Scale bar = 50 µm. The images are representative of three biological repeats.

We previously showed that the newly developed C-5 carbamate sialyltransferase inhibitor P-SiaFNEtoc [82] can effectively inhibit the sialylation of prostate cancer cells with only minor effects on other glycan types [51]. Next, to test whether P-SiaFNEtoc can inhibit α2,6-sialylated *N*-glycans in enzalutamide-resistant prostate cancer cells, we treated VCaP^EnzR^ and LNCaP^EnzR^ cells with P-SiaFNEtoc and monitored α2,6-sialylation using SNA lectin immunofluorescence after 3 or 6 days (Supplementary Figure S2). For the VCaP^EnzR^ cells, treatment with 20 µM P-SiaFNEtoc for 6 days suppressed recognition by SNA lectin, suggesting a reduction in α2,6-sialylated *N*-glycans (Figure 3A). For the LNCaP^EnzR^ cells, treatment with 2 µM P-SiaFNEtoc for 3 days reduced levels of levels of α2,6 sialylation (Figure 3B). As VCaP cells have a significantly longer doubling time than LNCaP cells [92,93], and have higher endogenous levels of ST6GAL1 [87], we hypothesised that this may explain why the VCaP cells required increased concentrations of P-SiaFNEtoc to reduce α2,6-sialylation levels, but we did not investigate this further. Together with previously published findings [51], our data show that treatment with the sialyltransferase inhibitor P-SiaFNEtoc can block α2,6 sialylation in enzalutamide-resistant prostate cancer cells.

### 3.4. Sialic Acid Blockade Partially Reverts Acquired Resistance to Enzalutamide in Prostate Cancer Cells

**Figure 4:**
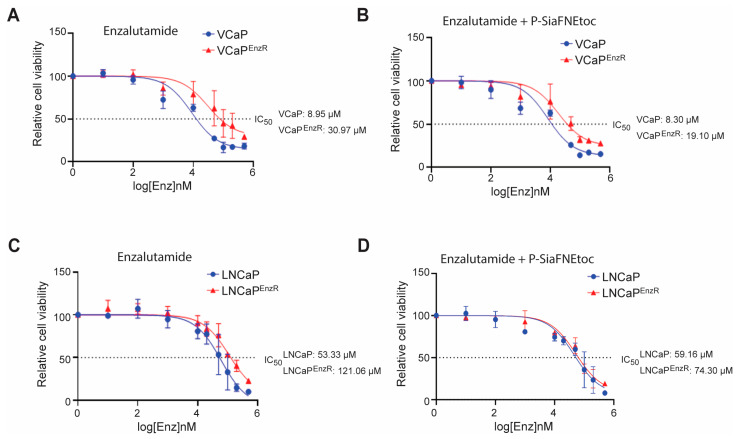
Sialic acid blockade using P-SiaFNEtoc partially re-sensitises prostate cancer cells to the second-generation androgen receptor antagonist enzalutamide. (**A**) The VCaP^EnzR^ cells have increased resistance to enzalutamide compared to the control VCaP cells. The control VCaP and VCaP^EnzR^ cells were treated with a range of enzalutamide concentrations (0–500 µM). Cell viability was measured after 6 days using a CellTitrer-Glo^®^ luminescence assay. The IC_50_ values (the concentration of enzalutamide which reduced cellular viability by 50% relative to the DMSO control) was 3.46-fold greater in the VCaP^EnzR^ cells (8.95 µM for VCaP control cells and 30.97 µM for the VCaP^EnzR^ cells). (**B**) Treatment of the VCaP^EnzR^ cells with P-SiaFNEtoc partially reverts resistance to enzalutamide. The control VCaP and VCaP^EnzR^ cells were treated with 20 µM P-SiaFNEtoc and a range of concentrations of enzalutamide (0–500 µM) for 6 days. Inhibiting sialylation in the VCaP^EnzR^ cells reduced the IC_50_ value from 30.97 µM to 19.10 µM, indicating a partial reversion of their resistance to enzalutamide. (**C**) The LNCaP^EnzR^ cells have increased resistance to enzalutamide compared to the control LNCaP cells. The control LNCaP and LNCaP^EnzR^ cells were treated with a range of enzalutamide concentrations (0–500 µM). Cell viability was measured after 3 days using a CellTitre-Glo^®^ luminescence assay. The IC_50_ value was 2.27-fold higher in the LNCaP^EnzR^ cells (53.33 µM for LNCaP control cells and 121.06 µM for the LNCaP^EnzR^ cells). (**D**) Treatment of the LNCaP^EnzR^ cells with P-SiaFNEtoc partially reverts their resistance to enzalutamide. The control LNCaP and LNCaP^EnzR^ cells were treated with 2 µM P-SiaFNEtoc and a range of concentrations of enzalutamide (0–500 µM) for 3 days. A DMSO-only control arm was included for each cell line. Inhibiting sialylation in the LNCaP^EnzR^ cells reduced the IC_50_ value from 121.06 µM to 74.30 µM, indicating a partial reversion of their resistance to enzalutamide. A line of best fit was utilised for interpolating the IC_50_ value. Results are presented as the cell viability (luminescence) relative to the respective DMSO control against the log of the enzalutamide concentration (nM) used for each cell line. Results are presented as the mean ± standard error and are representative of three biological repeats.

Together with the literature [59,60,61,62,63,64,65,66,67,68,69,70,71,72], the above data raises the possibility that aberrant sialylation could play a functional role in the acquired resistance of prostate cancer to the second-generation AR antagonist enzalutamide. This led to the hypothesis that therapies targeting sialylation might have the potential to re-sensitise prostate cancer cells to treatment with enzalutamide. To investigate this, we first treated control VCaP and VCaP^EnzR^ cells with a range of enzalutamide concentrations (up to 500 µM) and measured cellular viability using CellTiter-Glo^®^ luminescence assays and IC_50_ analyses. As expected, the IC_50_ value for the VCaP^EnzR^ cells treated with enzalutamide was 3.46-fold higher relative to the control VCaP cells (VCaP control IC_50_: 8.95 µM, VCaP^EnzR^ IC_50_: 30.97 µM) (Figure 4A). Next, to test if sialic acid blockade can impact the resistance of prostate cancer cells to enzalutamide, we treated the VCaP^EnzR^ cells with enzalutamide in combination with the sialylation inhibitor P-SiaFNEtoc (treatments for VCaP cells were carried out with 20 µM P-SiaFNEtoc for 6 days to match our findings in Figure 3A). Excitingly, this revealed that although sialic acid blockade in combination with enzalutamide therapy did not alter the IC_50_ value for the VCaP control cell line, for the VCaP^EnzR^ cells, there was a 38.33% reversion in the IC_50_ value in comparison to enzalutamide treatment alone (VCaP control IC_50_: 8.30 µM, VCaP^EnzR^ IC_50_: 19.10 µM) (Figure 4B). 

The IC_50_ value for the LNCaP^EnzR^ cells was 2.27-fold higher relative to the control LNCaP cells when treated with enzalutamide alone (LNCaP control: 53.33 µM, LNCaP^EnzR^: 121.06 µM) (Figure 4C). When the LNCaP^EnzR^ cells were treated with both P-SiaFNEtoc and enzalutamide (treatments for the LNCaP cells were carried out with 2 µM P-SiaFNEtoc for 3 days to match our findings in Figure 3B), there was a 38.64% reversion in the IC_50_ value in comparison to enzalutamide treatment alone (LNCaP control IC_50_: 59.16 µM, LNCaP^EnzR^ IC_50_: 74.30 µM) (Figure 4C,D). These data indicate that that sialic acid blockade, in combination with enzalutamide treatment, can partially revert acquired enzalutamide resistance in cell line models of prostate cancer.

## 4. Discussion

Enzalutamide is an orally administered small-molecule inhibitor of the AR that is designed to overcome resistance to anti-androgens and can improve overall survival in CRPC [19,20,94]. Unfortunately, some patients have primary resistance to enzalutamide, and others eventually develop acquired resistance and continue to progress [27,95]. Numerous studies have sought to characterise the molecular mechanisms underlying how prostate cancer becomes resistant to enzalutamide therapy, with AR amplification, AR variants, altered expression of AR co-regulators, upregulation of glucocorticoid receptor (GR), and metabolic alterations believed to play a role [95,96]. ST6GAL1 and its associated glycans are likely to be an important target in cancer cells [89,90]. ST6GAL1 is upregulated in numerous types of cancer, including pancreatic, ovarian, breast, and prostate cancer, and its expression is associated with aggressive tumours and reduced survival times [52,89,97,98]. Upregulation of ST6GAL1 impacts oncogenic cell behaviours and can play a key role in tumour growth, survival, metastasis, immune evasion, and resistance to therapy [89,97,99]. Specifically, ST6GAL1 can mediate resistance to chemoradiation in rectal cancer [73], the sensitivity of gastric cancer cells to trastuzumab [72], and resistance to the EGFR inhibitor, gefitinib, in ovarian cancer [100]. In prostate cancer, the upregulation of ST6GAL1 promotes the growth and metastatic spread of prostate tumours to bone [52,77], and targeting aberrant sialylation in prostate cancer cells can inhibit the metastatic spread of tumours to bone [51,52]. The data presented in this manuscript indicate that ST6GAL1-mediated aberrant sialylation could also be an important mediator of the acquired resistance of prostate tumours to enzalutamide therapy (which is a major clinical issue potentially affecting all men who develop CRPC [101]. Furthermore, our study provides proof-of-concept data for a therapeutic strategy to target aberrant sialylation that has the potential to partially revert enzalutamide resistance and prolong the clinical efficacy of anti-androgen therapies. It should be noted that the sialyltransferase inhibitor used in this study (P-SiaFNEtoc) is a global sialyltransferase inhibitor that targets all sialyltansferase enzymes (including ST6GAL1), meaning we cannot rule out that that the effects of P-SiaFNEtoc on the enzalutamide-resistant cells may not be solely due to ST6GAL1 inhibition. Inhibitors specifically targeting ST6GAL1 are being developed [56,102] and, once available, will be highly relevant to prostate cancer.

The mechanisms underpinning how aberrant sialylation might contribute to enzalutamide resistance in prostate cancer remain unclear but are likely multi-faceted. Aberrant sialylation on the cell surface could potentially inhibit the uptake of enzalutamide into the cell. Another possibility is that alterations to sialylated glycans could interfere with the binding of enzalutamide to the AR to promote resistance. The role of sialic acid in the context of the tumour microenvironment and immune suppression is also an important consideration which was not addressed by the current study. Increased sialylation is common in cancer cells and is associated with an immunosuppressed tumour microenvironment [57,99,103,104]. Sialylated glycans can be engaged by a broad range of immune cell types to promote immune suppression [105,106], and the upregulation of ST6GAL1 in prostate cancer cells has been shown to promote a shift towards an immunosuppressive M2 macrophage phenotype [52]. Anti-androgen therapies are known to remodel the prostate tumour immune microenvironment [107,108], and, recently, it was proposed that this remodelling could include alterations to the Siglec–sialyloglycan axis in prostate tumours, which could contribute to immunosuppression [109]. Moving forward, syngeneic models of prostate cancer could be used to perform pre-clinical evaluation of sialic acid blockade in combination with enzalutamide therapy, alongside investigating the role of altered glycosylation in the resistance to enzalutamide therapy in the context of the tumour-immune microenvironment. Previous studies have identified increased levels of ST6GAL1 in tumours and blood samples from prostate cancer patients [52,77]. In future studies, it will be important to monitor the levels of ST6GAL1 and sialylated glycans in clinical samples from prostate cancer patients with tumours that have become resistant to enzalutamide therapy and investigate whether the detection of ST6GAL1 and/or aberrant sialylation in prostate tumours could be used to predict sensitivity and resistance to treatment strategies.

## 5. Conclusions

Our findings identify aberrant sialylation as a previously unexplored but clinically relevant contributor to the acquired resistance of prostate tumours to enzalutamide therapy and suggest further research in this area could lead to the development of novel combination therapies to disarm drug resistance in prostate cancer. Recent studies have provided rationale for the use of glyco-immune checkpoint-targeting therapies in advanced prostate cancer [52,109]. Numerous strategies to target sialylated glycans are under development, including sialylation inhibitors and antibody–sialidase conjugates [34,50,110], some of which are currently in clinical trials [34,50] and are likely to be relevant for patients with prostate cancer. Future research should seek to utilise these advancements in the glycobiology field to gain a better understanding of the role which aberrant glycosylation may play in prostate cancer resistance to key therapeutic interventions, such as enzalutamide.

## Data Availability

The authors confirm that the data supporting the findings of this study are available within the article and its Supplementary Materials.

## Supplementary Materials

The following supporting information can be downloaded at: https://www.mdpi.com/article/10.3390/cancers16172953/s1, Figure S1: SNA binding was eliminated when prostate cancer cells were treated with neuraminidase; Figure S2: Detection of α2,6 sialylation in VCaP cells treated with 2–20 µM P-SiaFNEtoc. File S1: Original western blots.
