# Supplementary material for: Sialylation Inhibition Can Partially Revert Acquired Resistance to Enzalutamide in Prostate Cancer Cells
Source: Cancers (Basel). 2024 Aug 24;16(17):2953. doi: 10.3390/cancers16172953 (PMC11393965; doi:10.3390/cancers16172953)

Supplementary Figure S1
SNA binding wass eliminated when prostate cancer cells were treated with neuraminidase (which removes sialic acids from glycans)

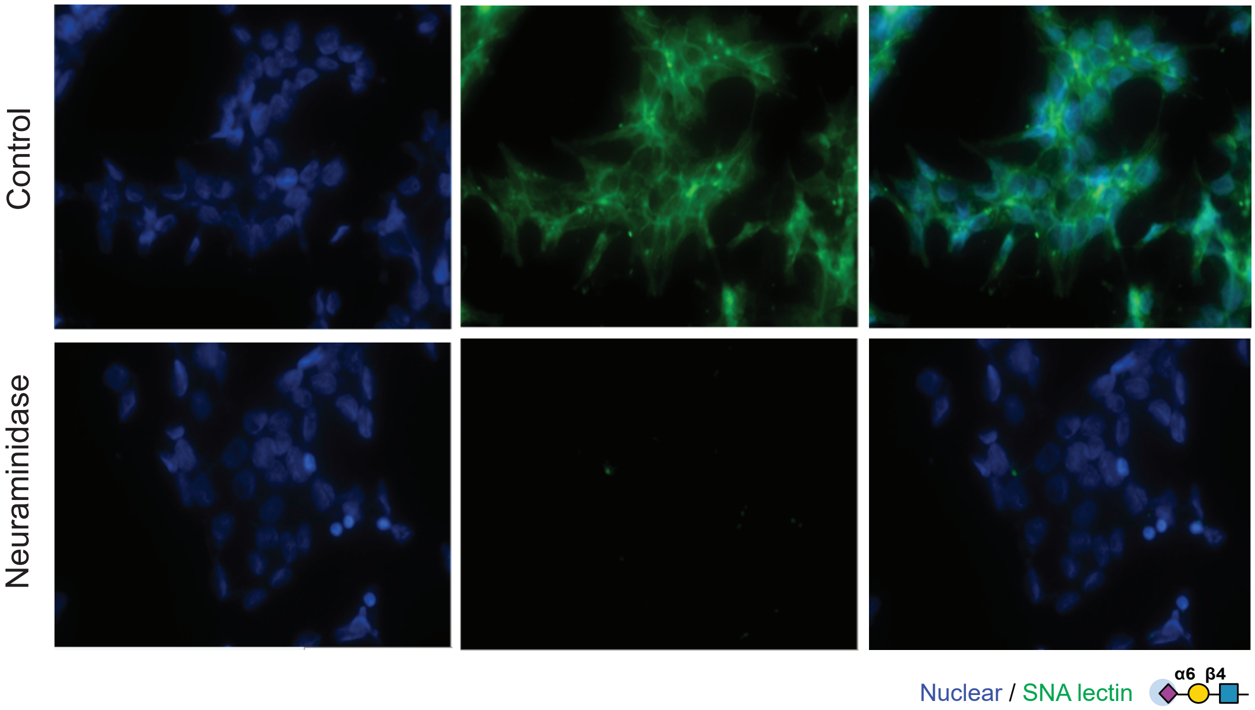

## Supplementary Figure S2

Detection of immunofluorescent staining of ST6GAL1 and α2,6-sialylation of N-glycans in VCaP control and VCaPEnzR cells treated with 2, 10 and 20 μM of the sialyltransferase inhibitor P-SiaFNEtoc for 6 days. Treatment of both cell lines with 20 μM P-SiaFNEtoc inhibits α2,6-sialylation of N-glycans (de-tected using SNA lectin).

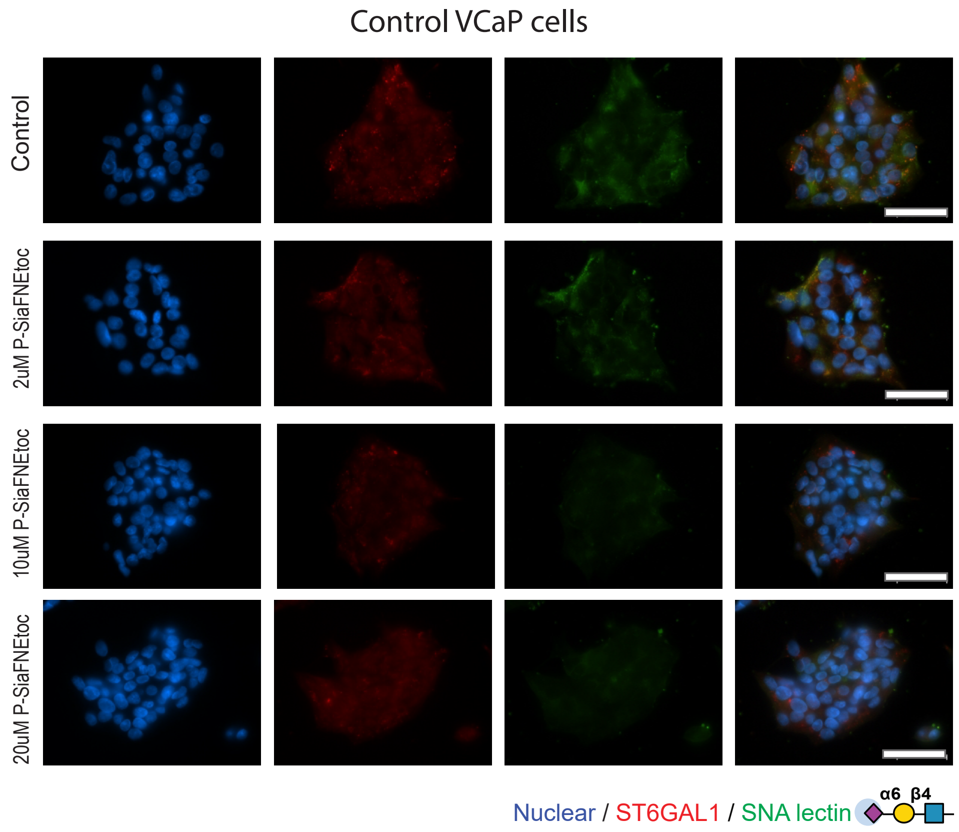


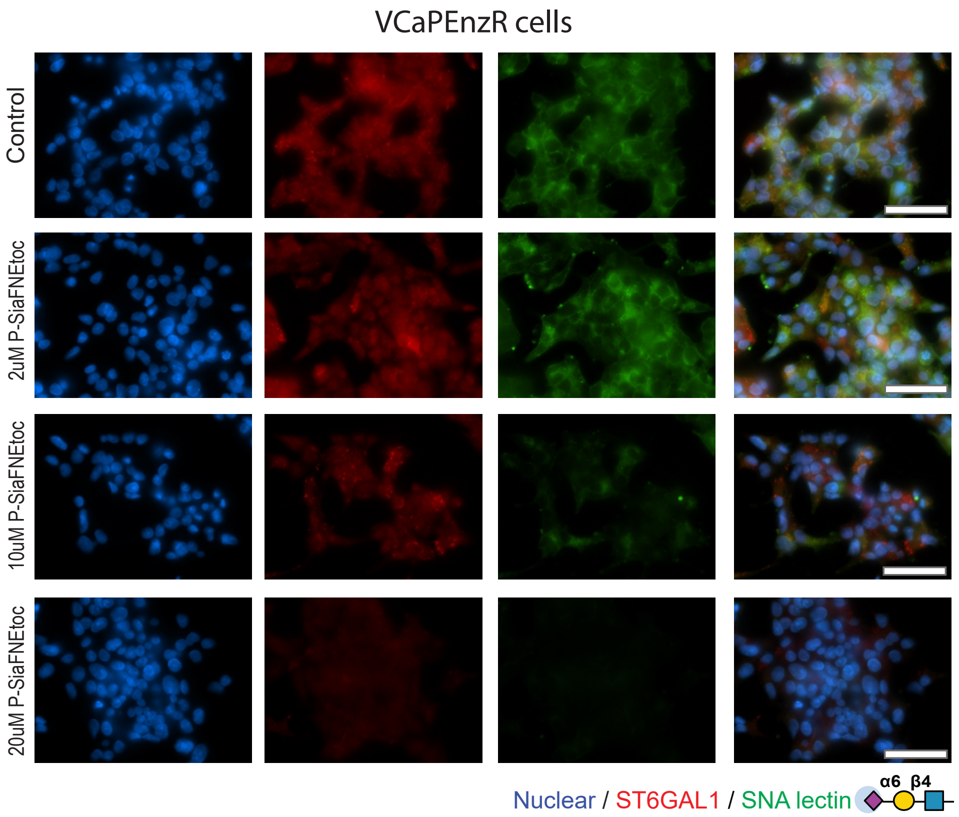

Supplement: Supplementary file 1 [file cancers-16-02953-s001.zip › cancers-3138686-supplementary.pdf]
