# Supplementary figures and images for: Sialylation Inhibition Can Partially Revert Acquired Resistance to Enzalutamide in Prostate Cancer Cells
Source: Cancers (Basel). 2024 Aug 24;16(17):2953. doi: 10.3390/cancers16172953 (PMC11393965; doi:10.3390/cancers16172953)

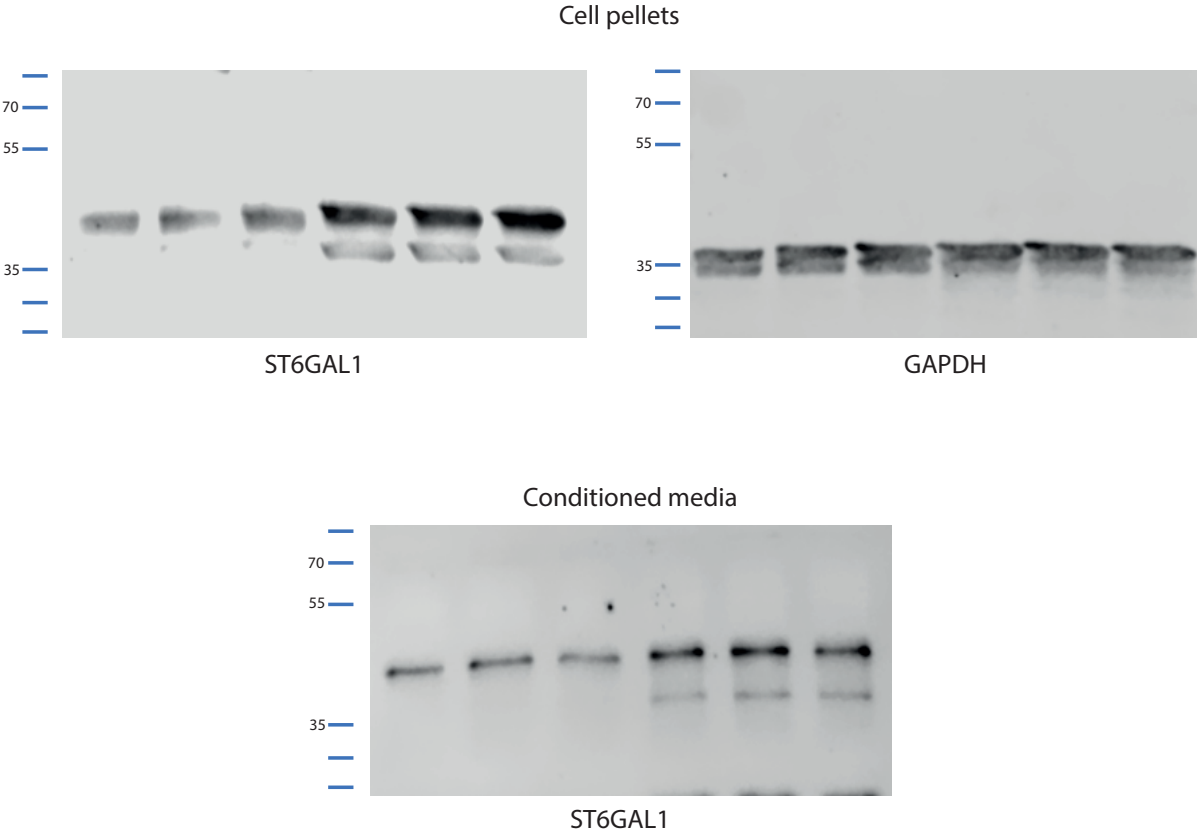

Supplement: Supplementary file 1 [file cancers-16-02953-s001.zip › cancers-3138686-File S1.pdf]
